# Automating untruths: ChatGPT, self-managed medication abortion, and the threat of misinformation in a post-*Roe* world

**DOI:** 10.3389/fdgth.2024.1287186

**Published:** 2024-02-14

**Authors:** Hayley V. McMahon, Bryan D. McMahon

**Affiliations:** ^1^ Department of Behavioral, Social, and Health Education Sciences, Emory University Rollins School of Public Health, Atlanta, GA, United States; ^2^ The Center for Reproductive Health Research in the Southeast, Emory University Rollins School of Public Health, Atlanta, GA, United States; ^3^ Independent Researcher, Atlanta, GA, United States

**Keywords:** self-managed abortion, generative artificial intelligence, ChatGPT, health misinformation, reproductive health, health communication

## Abstract

**Background:**

ChatGPT is a generative artificial intelligence chatbot that uses natural language processing to understand and execute prompts in a human-like manner. While the chatbot has become popular as a source of information among the public, experts have expressed concerns about the number of false and misleading statements made by ChatGPT. Many people search online for information about self-managed medication abortion, which has become even more common following the overturning of *Roe v. Wade*. It is likely that ChatGPT is also being used as a source of this information; however, little is known about its accuracy.

**Objective:**

To assess the accuracy of ChatGPT responses to common questions regarding self-managed abortion safety and the process of using abortion pills.

**Methods:**

We prompted ChatGPT with 65 questions about self-managed medication abortion, which produced approximately 11,000 words of text. We qualitatively coded all data in MAXQDA and performed thematic analysis.

**Results:**

ChatGPT responses correctly described clinician-managed medication abortion as both safe and effective. In contrast, self-managed medication abortion was inaccurately described as dangerous and associated with an increase in the risk of complications, which was attributed to the lack of clinician supervision.

**Conclusion:**

ChatGPT repeatedly provided responses that overstated the risk of complications associated with self-managed medication abortion in ways that directly contradict the expansive body of evidence demonstrating that self-managed medication abortion is both safe and effective. The chatbot's tendency to perpetuate health misinformation and associated stigma regarding self-managed medication abortions poses a threat to public health and reproductive autonomy.

## 1 Introduction

On June 24, 2022, the Supreme Court of the United States ruled in *Dobbs v. Jackson Women's Health Center* (*Dobbs*) to overturn its 1973 landmark decision *Roe v. Wade* (*Roe*), which had established a federal right to abortion. Many communities were already experiencing crises in abortion access long before the *Dobbs* ruling, but the devastating fallout of the decision has only further decimated access to clinician-managed abortion care across the entire country. In just the first nine months after *Dobbs*, researchers estimate that over 25,000 people were denied clinician-managed abortion care (1). More than twenty states have now enacted gestation-based abortion bans, forcing at least 66 former abortion clinics to end abortion services and leaving entire subregions in the South and Midwest without any access to clinician-managed abortion care (2, 3). Meanwhile, in states with legal abortion, most clinics are overwhelmed by the enormous influx of out-of-state patients. While many people simply cannot afford to make the incredibly expensive and disruptive trip, even those who do have the resources to travel hundreds or thousands of miles from a legally restricted state must often remain pregnant for weeks while they wait to be seen (4). These worsening delays and denials are particularly concerning for Black, Indigenous, Latinx, and/or low-income people, who simultaneously experience the greatest need for abortions and the poorest access to clinician-managed abortion care (5–7).

While it is certainly not a feasible or acceptable option for everyone, many pregnant people who cannot or do not wish to access clinician-managed abortion care instead choose to self-manage their abortion, often by using pills (8). Self-managed medication abortion (SMMA) refers to the process by which a pregnant person obtains and uses mifepristone and misoprostol or misoprostol alone to end a pregnancy without the direct involvement of a clinician. In some cases, the individual may ultimately choose to seek follow-up care or medical advice from a clinician, but it is the pregnant person and not the clinician who procures abortion pills outside of the formal healthcare system and administers them on their own terms. Beyond this element of autonomous management, little else separates SMMA from clinician-managed medication abortion—both follow the same medication protocols and have the same high rates of safety and effectiveness (9). SMMA was already quite common before *Roe v. Wade* was overturned, and post-*Dobbs* data suggests it has only become more prevalent since then (8, 10). However, the stigma and looming threat of criminalization surrounding SMMA lead many people to discretely seek information about abortion pills online. This source of information is of particular importance for people of color, low-income people, and those living in the Southeastern U.S., who more frequently utilize SMMA and face a disproportionate burden of criminalization (8–14). Unfortunately, research has shown that Google search results are rife with abortion misinformation, making it incredibly difficult for people to discern the truth (15). As such, developing innovative strategies to disseminate accurate information about SMMA has been a critical priority of post-*Roe* health promotion.

Just five months after the *Dobbs* ruling came down, a California start-up called OpenAI publicly launched its generative artificial intelligence (AI) chatbot. ChatGPT is a conversational chatbot that interprets and responds to text prompts in real time, enabling it to answer questions, develop computer code, or write essays in a human-like manner. ChatGPT quickly went viral, gaining more than 100 million daily users within two months and easily surpassing growth records (16). Even after this initial surge slowed, ChatGPT still sees more than 1.5 billion users per month (17). Its widespread uptake and diverse applicability prompted a buzz among many health professionals, many of whom view ChatGPT as a promising tool for health education (18, 19). In particular, the potential accessibility and privacy offered by a well-known chatbot providing timely and reliable responses could be invaluable to efforts to educate the public on highly stigmatized health issues like SMMA. However, numerous instances of ChatGPT providing misleading, incorrect, and even wildly fabricated responses have been documented (20, 21). The chatbot operates using the Generative Pre-Trained Transformer algorithm, which relies on natural language processing to understand and execute commands. Unlike search engines that locate and return existing results, ChatGPT parses human prompts and predicts strings of words to generate a response, which allows it to create novel material. The algorithm is also nondeterministic, meaning that the accuracy of its statements cannot be predicted (22–24).

Given ChatGPT's immense popularity, it is undoubtedly being used to locate health information. A growing body of literature has documented concerning instances of the chatbot generating replies that contain health misinformation, but ChatGPT responses on SMMA have yet to be explored (25–28). At a time when more and more Americans are opting to self-manage their abortions at home with pills, it is critical that we understand the reliability of information being provided by the immensely popular chatbot. Thus, the current study aimed to assess the accuracy of ChatGPT's responses to fundamental questions regarding SMMA safety and the general process of using abortion pills.

## 2 Methods

### 2.1 Reflexivity

HVM is a public health scientist with a primary research focus on abortion misinformation and has substantial experience and graduate-level training in qualitative research methods. BDM is a technology research analyst who specializes in artificial intelligence. In order to practice bracketing and limit the impact of *a priori* assumptions from their respective fields of expertise, both authors independently worked on both the abortion and AI elements of the study.

### 2.2 Data collection

We began data collection by developing a semi-structured interview guide consisting of 65 basic questions about medication abortion across three core domains: (1) medication abortion protocols, (2) common experiences during medication abortion, and (3) the safety of self-managed medication abortion. To limit the need for a more subjective interpretation of accuracy by the researchers, questions were intentionally developed to have straightforward answers that are well established in the scientific literature. Table 1 presents 15 examples of items from the interview guide. We posed each question from the guide to ChatGPT one at a time and probed further on any unclear answers. Given the repetition present in the data, both researchers felt that meaning saturation was reached (29).

**Table 1:** Examples of interview guide questions.

1. How do you use misoprostol pills for abortion?
2. What are the instructions for misoprostol-only abortion?
3. How does mifepristone work in an abortion?
4. Are abortion pills safe?
5. What are the risks of using abortion pills?
6. How do you care for yourself during a medication abortion?
7. What are the normal side effects of abortion pills?
8. How much bleeding is normal after using abortion pills?
9. How do you know a medication abortion worked?
10. Can people have a safe medication abortion at home without a clinician?
11. What is self-managed abortion with pills?
12. How does self-managed abortion with pills work?
13. How safe is self-managed abortion with pills?
14. How common are complications of self-managed abortion using pills?
15. Where do people get pills for self-managed abortion?

At the time of data collection, ChatGPT used the Generative Pre-Trained Transformer-3.5 language model. Because this version of the chatbot does not incorporate new information in real time and is unable to access information posted on the internet after 2021, it was not necessary to repeat this process at multiple time points or with multiple devices (23). We exported all responses to a single document, which consisted of approximately 11,000 total words. This study did not involve human subjects.

### 2.3 Analysis

Thematic analysis was conducted according to Braun and Clarke's six-phase protocol: “Familiarizing yourself with the data, generating initial codes, searching for themes, reviewing themes, defining and naming themes, and producing the report” (30). We first became familiar with the data by reviewing and annotating all exported responses. Based on these annotations, we inductively developed and refined a codebook based on patterns identified in the data. This was used to code all data in MAXQDA, a computer-assisted qualitative data analysis software (CAQDAS). We then produced matrices of text segments by code in order to develop initial themes. Thematic mapping was employed to iteratively refine themes and their relationships, which prompted us to create additional data matrices that facilitated comparison of ChatGPT responses regarding clinician-managed medication abortion vs. SMMA. Finally, thematic maps were validated and refined through post-hoc concept-indicator modelling and investigator triangulation (31, 32).

## 3 Results

Three core themes emerged throughout our analysis of ChatGPT's responses: (1) the provision of accurate information regarding the use of abortion pills (2) the continuous emphasis on clinician management as a requirement for safe use of medication abortion and (3) the frequent overstatement of potential risks associated with SMMA.

### 3.1 Use of abortion pills

ChatGPT produced mostly accurate responses regarding the general process of using abortion pills. Although the chatbot repeatedly provided a disclaimer that it “…cannot provide specific medical advice or instructions on how to use medication,” ChatGPT still correctly described the mifepristone-misoprostol and misoprostol-only regimens for inducing an abortion using pills. It also accurately represented common, short-term effects of the medications: “The most common side effects include cramping, nausea, vomiting, diarrhea, and bleeding, which are normal parts of the abortion process” (33). Notably, ChatGPT did provide a somewhat misleading response regarding the administration of misoprostol: “When used for medical abortion, misoprostol is typically taken orally or inserted into the vagina.” Rather than taking misoprostol orally, most clinicians recommend dissolving the tablets buccally, sublingually, or vaginally as these routes of administration are more effective (34).

### 3.2 Clinician management

While ChatGPT correctly defined SMMA as “the process of ending a pregnancy without direct involvement from a healthcare provider,” it repeatedly emphasized that SMMA should only be done with medical supervision—a clear contradiction to the concept of autonomous self-management. Information was presented in a way that implied safety was conditional on clinician management: “Abortion pills are considered safe and effective for ending an early pregnancy when taken under the supervision of a healthcare provider and after a thorough medical evaluation.” Many of these responses did not reflect the substantial body of literature demonstrating that the safety and effectiveness of abortion pills remains unchanged by forgoing clinician involvement in favor of self-management: “Having a medication abortion at home without a clinician is not recommended. While medication abortion can be a safe and effective method for ending a pregnancy when done under medical supervision, it is important to have the guidance and support of a healthcare provider throughout the process” (9, 35).

### 3.3 Safety of self-managed medication abortion

In addition to the continuous emphasis on the necessity of clinician management, ChatGPT's responses frequently overstated the potential risks of SMMA: “It is important to emphasize that self-managed abortion with pills is not recommended due to the potential risks and dangers involved. Seeking medical care from a qualified healthcare provider is always the safest approach when considering an abortion.” When prompted with follow-up questions about the risks, ChatGPT again drew on clinician management in a way that directly contradicts existing evidence: “Attempting a medication abortion without the involvement of a healthcare provider increases the risk of incomplete abortion, inadequate management of side effects or complications, and other potential health risks” (35, 36).

The chatbot also misrepresented concerns regarding ectopic pregnancy in relation to SMMA, stating that, “If a person has an ectopic pregnancy, medication abortion with pills will not be effective and may cause serious complications” and “In rare cases, the medication may not work as intended and an ectopic pregnancy may result.” ChatGPT correctly noted that abortion pills are not effective for treating ectopic pregnancies; however, the other claims in these statements are untrue. Abortion pills do not affect and certainly do not cause ectopic pregnancies. Some clinicians have expressed concerns that self-managed medication abortion could lead to delays in detecting ectopic pregnancies; however, research does not support this (37, 38).

## 4 Discussion

Given the immense popularity of ChatGPT and frequency with which people turn to the internet for information on SMMA, it is highly probable that ChatGPT will be serve as a common source for locating information about SMMA in the post-*Roe* era (13, 14). Our findings on ChatGPT's ability to accurately respond to fundamental questions regarding the use of abortion pills and the safety of SMMA were mixed. Overall, the chatbot performed well in terms of responding to prompts about the proper use of abortion pills. It also accurately depicted normal side effects that a person could expect to experience throughout the abortion process, and it may be useful in this regard. Concerningly, ChatGPT was much less reliable when queried about the safety of SMMA. The chatbot significantly misrepresented the importance of clinician management and overstated the potential health risks of SMMA.

Just as with any medication, mifepristone and misoprostol carry a small chance of complications, and this information should certainly be readily available to those who are considering SMMA. However, infection, heavy bleeding, and incomplete abortion—all incorrectly presented by ChatGPT as increased risks associated with SMMA—are exceptionally rare and have relatively straightforward treatments, such as prescribing oral antibiotics or performing a procedural abortion that uses gentle suction to remove retained tissue (39). Yet, ChatGPT's responses contradict evidence that was available online well before the chatbot's training material cutoff date in late 2021. By 2015, for example, the World Health Organization (WHO) had deemed SMMA to be safe, effective, and acceptable (40, 41). In 2020, WHO also published its own “WHO Recommendations on Self-Care Interventions: Self-Management of Medical Abortion” guidance document, which includes an evidence based SMMA protocol (42). Furthermore, researchers have found that SMMA with mifepristone and misoprostol is approximately 96% effective with fewer than 1% of people experiencing a serious adverse event. Studies have also established that the risk of complications does not significantly vary between clinician-managed medication abortions and SMMA (35, 36). This holds true for ectopic pregnancies as well; the data does not show a delay in recognizing an ectopic pregnancy or receiving appropriate care among those who choose SMMA (35). Therefore, it is concerning that ChatGPT's responses convey a sense of peril in regard to SMMA by characterizing it as “dangerous,” “not recommended,” and a method that “carries an increased risk of complications,” none of which is supported by the extensive body of available evidence.

As such, our findings demonstrate it is likely that ChatGPT is disseminating misinformation about SMMA, which can promote unnecessary fear and stigma. In turn, abortion stigma elicits emotional distress and can push pregnant people toward truly unsafe methods, such as using toxic substances or physical trauma to end a pregnancy (12, 43). Furthermore, these unfounded fears can leave those who cannot or do not wish to access clinician-managed abortion care feeling as if they have no choice but to carry an undesired or mistimed pregnancy to term. Longitudinal research has shown that such an experience is associated with a range of negative outcomes, including an increased risk of living in poverty, remaining in contact with an abusive partner, suffering from chronic illnesses, and experiencing poor parental bonding (44).

Although continued refinement of generative AI technology like ChatGPT is expected, it is imperative that health educators, advocates, and software engineers are aware of the present shortcomings of ChatGPT and their potential implications for health. The field of generative AI is rapidly expanding, and ChatGPT is likely not alone in its tendency to provide false information about SMMA or other health issues. Google, for example, has also announced that it will integrate its own generative AI chatbot into Google Search, so the same health misinformation concerns posed by ChatGPT may soon seep into the world's most popular search engine (45). The fact remains that nondeterministic algorithms like ChatGPT that piece together strings of word without predictable accuracy are ultimately destined to generate misinformation—and an application with more than 1.5 billion monthly users that, in its current state, exaggerates the risks of SMMA poses a threat to bodily autonomy and to public health.

## Data Availability

The raw data supporting the conclusions of this article will be made available by the authors, without undue reservation.
